# What do bereaved parents want from professionals after the sudden death of their child: a systematic review of the literature

**DOI:** 10.1186/1471-2431-14-269

**Published:** 2014-10-15

**Authors:** Joanna Garstang, Frances Griffiths, Peter Sidebotham

**Affiliations:** Division of Mental Health and Wellbeing, Warwick Medical School, Coventry, CV4 7AL UK; Division of Health Sciences, Warwick Medical School, Coventry, CV4 7AL UK

**Keywords:** Child death, Bereavement, Parent, Sudden infant death syndrome, Child death review, Multi-professional working, Physician interactions

## Abstract

**Background:**

The death of a child is a devastating event for parents. In many high income countries, following an unexpected death, there are formal investigations to find the cause of death as part of wider integrated child death review processes. These processes have a clear aim of establishing the cause of death but it is less clear how bereaved families are supported. In order to inform better practice, a literature review was undertaken to identify what is known about what bereaved parents want from professionals following an unexpected child death.

**Methods:**

This was a mixed studies systematic review with a thematic analysis to synthesize findings. The review included papers from Europe, North America or Australasia; papers had to detail parents’ experiences rather than professional practices.

**Results:**

The review includes data from 52 papers, concerning 4000 bereaved parents. After a child has died, parents wish to be able to say goodbye to them at the hospital or Emergency Department, they would like time and privacy to see and hold their child; parents may bitterly regret not being able to do so. Parents need to know the full details about their child’s death and may feel that they are being deliberately evaded when not given this information. Parents often struggle to obtain and understand the autopsy results even in the cases where they consented for the procedure. Parents would like follow-up appointments from health care professionals after the death; this is to enable them to obtain further information as they may have been too distraught at the time of the death to ask appropriate questions or comprehend the answers. Parents also value the emotional support provided by continuing contact with health-care professionals.

**Conclusion:**

All professionals involved with child deaths should ensure that procedures are in place to support parents; to allow them to say goodbye to their child, to be able to understand why their child died and to offer the parents follow-up appointments with appropriate health-care professionals.

**Electronic supplementary material:**

The online version of this article (doi:10.1186/1471-2431-14-269) contains supplementary material, which is available to authorized users.

## Background

The death of a child can be considered one of the most devastating life events for parents. It is an upset to the natural order of events; most parents rightly expect their children to outlive them. Several years after their child has died, bereaved parents may continue to feel the impact of the death on a daily basis [1].

The investigation following an unexpected child death varies in different countries, but in many cases involves the police or Coroner as well as health services, with many now having integrated Child Death Review (CDR) processes [2]. CDR typically includes overview of child deaths at a population level with the intention of learning lessons and preventing future deaths; a process that rarely involves parents. However, in many countries CDR also involves detailed investigation of individual child deaths, requiring full medical and social histories from parents, death scene analysis and autopsy [3, 4]. This detailed CDR process has a clear focus on determining the cause of death but does not necessarily address the needs of the family; this is particularly pertinent as detailed CDR could be considered intrusive for the recently bereaved parents.

We undertook this literature review to inform best practice in supporting parents after sudden child death given the potential for intrusion of the new detailed CDR processes. We therefore reviewed the literature on bereaved parents’ interactions with professionals such as physicians, nurses, police officers and social workers after the death of their child. This review details bereaved parents’ experiences with such professionals and how the parents wished that they had been treated by professionals rather than professional opinions of what best care for bereaved parents may be. The question for our review is ‘What do bereaved parents want from professionals after the unexpected death of their children?’

## Methods

We used thematic analysis [5] and a narrative synthesis process [6].

The project did not require ethical approval as it only involved reviewing literature.

### Search strategy

#### Databases

We searched ASSIA, Ovid (MEDLINE) and CINAHL databases from 1.1.90 to 31.8.13. Google scholar was also used but limited to the first 10 screens of results only. We snowball searched all included articles. The search terms used are shown in Table 1.

**Table 1:** **Search terms used for literature searches**

Database	Search terms	
**Assia**	1	Child* and death or autopsy and parent* or bereavement and social worker or police or physician
	2	Child* and death and police or social work
	3	Child* and murder and parent*
**Ovid**	1	Grief or self-help group or prof- family relations or bereaved parent as keyword (k.w) And SIDS or child mortality or infant mortality or cause of death
	2	Death (expl- explode) – limit to <18 yrs And Bereavement expl/grief expl/parent# bereavement (k.w) And Autopsy expl
	3	Death expl – limit to <18 yrs And Bereavement expl/grief expl/parent# bereavement (k.w)/parent# expl And Forensic pathology expl/ forensic science expl/forensic# (k.w)/forensic medicine expl
	4	1. Death expl – limit to <18 yrs And Bereavement expl/grief expl/parent# bereavement (k.w)/ parent#expl And P?ediatrician (k.w) or physicians role expl or physician practice pattern exp or attitude of health personnel expl or physician expl or health visitor (k.w.) or community health nursing expl
	5	Death expl – limit to <18 yrs And Bereavement expl/grief expl/parent# bereavement (k.w) And Social worker (kw) or social work expl or police expl or police (kw)
	6	Death expl – limit to <18 yrs And coroner expl or medical examiner expl or coroner k.w.
**Cinhahl**		Search using ‘child death’ as word in abstract
**Google scholar**	1	‘bereaved parent doctor’
	2	‘bereaved parent social work’
	3	‘bereaved parent police’

#### Grey literature

We contacted or checked the websites of several bereavement associations and professional bodies for details of any unpublished research reports. These organisations were known to us from our practice or prior attendance at international conferences; they included The Lullaby Trust (UK), SIDS and Kids (Australia), International Society for the Study and Prevention of Infant Death (ISPID), the Child Bereavement Charity (UK), British Association for the Study and Prevention of Child Abuse and Neglect (BASCPAN, UK), Stillbirth and Neonatal Death Society (SANDS), Bereavement Care UK (Cruse) and Compassionate Friends UK.

#### Inclusion and exclusion criteria

We included original research and systematic reviews of research concerning bereaved parents interactions with health professionals, police or social workers. Included papers were from Europe, North America or Australasia and published from January 1990 to May 2014. Papers were included if they contained even minimal data on bereaved parents’ interactions with professionals even if this was not the main focus of the paper. We defined child death as death occurring from birth to 18 years. There was no limitation on language of publication.

We excluded papers concerning bereavement counselling as the sole interaction, papers with no data on liveborn children, papers containing data solely relating to children dying prior to 1980 and papers only concerning deaths by homicide or of terminally ill children.

### Selection process of studies and critical appraisal

JG read the titles, abstracts and full text articles twice (one month apart) for thoroughness. FG and PS reviewed a consecutive sample of 100 titles and abstracts for independent comparison. Formal translations were obtained for two non-English publications.

All included articles were critically appraised according to the overall nature (predominantly quantitative or qualitative) of the paper. We selected the critical appraisal tool for cross- sectional surveys [7] as it includes reference to development of the survey tools such as piloting and validation as well as sampling of the population. We used the critical appraisal tool for qualitative research [8] as it focuses on the key requirements available in most publications: appropriateness of the selected research methods; how participants were recruited; the relationship between the researcher and participants and methods of analyses.

### Data extraction, analysis and synthesis

We undertook data extraction and coding separately for quantitative and qualitative papers but used the same process. Firstly, we read the papers in their entirety then re-read them extracting relevant data. During extraction we developed and refined codes based on the data. All data was coded. Coded data was reviewed and codes from both qualitative and quantitative papers combined into themes.

However, the themes included data from studies that recruited bereaved parents whatever the cause of death and data from studies that recruited bereaved parents where the cause of death was of a distinct type such as neonatal death or SIDS. Many of the studies focused only on one aspect of the parent’s experience of child death. For our synthesis it was important to ensure we took account of this heterogeneity of studies.

We selected the data from two papers [9, 10] to create a reference framework against which data from the other studies could be compared. These data were chosen as together the papers from which they were extracted, reported studies that recruited parents experiencing all types of child death. Finlay and Dallimore [9] included any child death from any cause; Dent et al. [10] only included sudden deaths in children aged between 1 week and 12 years. They also studied all aspects of the experience including experiences at the time of the death in the Emergency Department, contact with the police and follow-up with General Practitioners and paediatricians. The process of synthesis involved comparison within each theme of the data from all other papers with the reference framework.

For each theme we present first the reference framework findings, and then we present our synthesis of data from other studies.

## Results

### Search results

**Figure 1:**
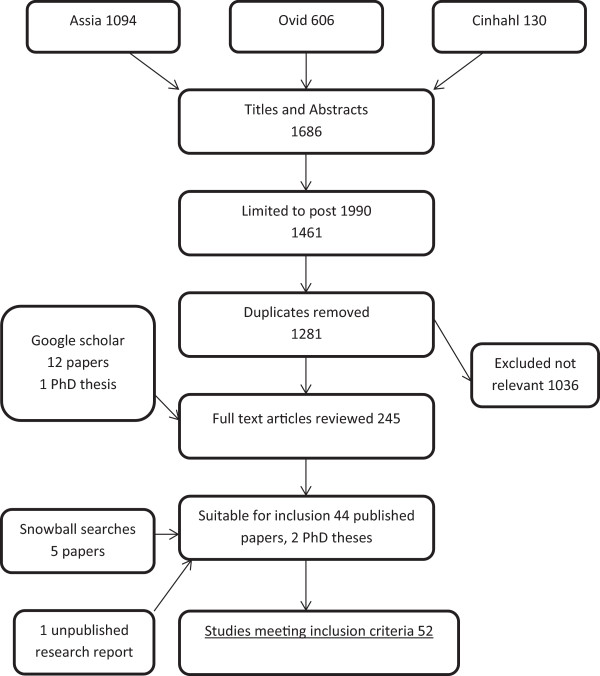
**Flow chart of selection process of papers.**

Out of 1294 titles and abstracts found by database searches 46 were suitable for inclusion. Snowball searching produced an additional 5 studies and we obtained 1 unpublished research report giving a total of 52 included studies, of which 25 were quantitative, 20 qualitative and 7 mixed. This is shown in Figure 1. More than 4000 bereaved parents participated in the original studies included in the review.

22 studies were from North America, 12 from the UK, 14 from other European countries and 4 from Australia. 19/25 quantitative studies were questionnaire surveys, 5 were interview surveys and one was a case series. 17/20 qualitative studies involved in-depth interviews with bereaved parents, 3 studies analysed data from open-ended questions in surveys. 4/7 mixed data studies were questionnaire studies, 2 were interview studies and 1 was a combination.

13 studies included child deaths from any cause, 16 studies were of perinatal deaths, 10 studies of SIDS, 7 studies were of deaths on paediatric intensive care units, 3 studies of deaths from trauma, 2 studies of deaths of children with intellectual disability and 1 of suicides. Most studies focused on bereaved parents experiences and perceptions of care and support or interaction with professionals; some purely focussed on views on autopsy.

Details of the included studies are given in Tables 2, 3 and 4.

**Table 2:** **Details of included quantitative studies**

Authors and year of publication	Name of study	Population and country	Study type	Proportion of study results included
W Ahrens, R Hart and N Maruyama [50] 1997	Pediatric death: managing the aftermath in the emergency department	SIDS parents	Postal questionnaire survey	100%
		N = 37		
		USA		
A Dent, L Condon, P Blair and P Fleming [10] 1996	A study of bereavement care after a sudden and unexpected death.	Parents of children dying suddenly	Postal questionnaire survey	50%
		N = 42		
		United Kingdom		
MA DiMarco, EM Menke and T McNamara [48] 2001	Evaluating a support group for perinatal loss	Parents of infants dying perinatally	Postal questionnaire survey	Minimal data
		N = 121		
		USA		
I Finlay and D Dallimore [9] 1991	Your child is dead	Parents of children dying of any cause at any age	Postal questionnaire survey	100%
		N = 120		
		United Kingdom		
MB Harper and NB Wisian [57] 1994	Care of bereaved parents. A study of patient satisfaction	Parents of infants dying perinatally and in infancy	Postal questionnaire survey or questionnaire distributed at support group	66%
		N = 28		
		USA		
A Hazzard, J Weston and C Gutterres [60] 1992	After a child's death: factors related to parental bereavement	Parents of children dying of any cause at any age	Structured interview	Minimal data
		N = 45		
		USA		
J Krauel Vidal, M Silva Vazquez, M Ibanez Fanes, R Florensa Palau and J Moreno Hernando [59] (translated from Spanish) 1992	Attitude towards parents after the death of their newborn infant in a neonatal unit	Parents of infants dying on neonatal units	Postal questionnaire survey	100%
		N = 49		
		Spain		
H Laakso and M Paunonen-Ilmonen [59] 2002	Mothers’ experience of social support following the death of a child	Mothers of children dying under age 7 years.	Questionnaire and structured interview	50%
		N = 91		
		Finland		
A Livesey [51] 2005	A multiagency protocol for responding to sudden unexpected death in infancy: descriptive study	Parents of infants dying suddenly and unexpectedly	Postal questionnaire survey as part of audit of practice	Minimal data
		N = 29		
		United Kingdom		
AJ Macnab, T Northway, K Ryall, D Scott and G Straw [23] 2003	Death and bereavement in a paediatric intensive care unit: Parental perceptions of staff support	Parents of children dying on intensive care unit	Questionnaire and structured interview	100%
		N = 24		
		Canada		
M McDonnell, A Cullen, B Kiberd, M Mehanni and T Matthews [52] 1999	A national model of care service for professionals dealing with sudden infant death	Parents of infants dying of SIDS	Structured interview	50%
		N = 131		
		Republic of Ireland		
EC Meyer, JP Burns, JL Griffith and RD Truog [56] 2002	Parental perspectives on end-of-life care in the pediatric intensive care unit	Parents of children dying on paediatric intensive care units	Postal questionnaire survey	33%
		N = 56		
		USA		
JR Neidig and P Dalgas-Pelish [25] 1991	Parental grieving and perceptions regarding health care professionals’ interventions	Parents of children dying of any cause at any age.	Postal questionnaire survey	25%
		N = 22		
		USA		
RC Oliver, JP Sturtevant, JP Scheetz and ME Fallat [33] 2001	Beneficial effects of a hospital bereavement intervention program after traumatic childhood death	Parents of children dying from trauma	Structured interview	Minimal data
		N = 54		
		USA		
BM Ostfeld, T Ryan, M Hiatt and T Hegyi [58] 1993	Maternal grief after sudden infant death syndrome	Parents of infants dying of SIDS	Postal questionnaire survey	Minimal data
		N = 38		
		USA		
M Powell [53] 1991	Sudden infant death syndrome: a crisis for parents and health professionals	Parents of infants dying of SIDS	Structured interview	25%
		N = 40		
		Republic of Ireland		
HA Rahman and TY Khong [45] 1995	Perinatal and infant postmortem examination. Survey of women's reactions to perinatal necropsy.	Mothers of infants dying perinatally	Telephone questionnaire survey	100% (published as letter only)
		N = 29		
		Australia		
J Rankin, C Wright and T Lind [43] 2002	Cross sectional survey of parents’ experience and views of the postmortem examination	Mothers of infants dying perinatally or in infancy	Postal questionnaire survey	100%
		N = 148		
		United Kingdom		
Royal College of Pathologists and Royal College of Paediatrics and Child Health [31] 2004	Sudden Unexpected Death in Infancy; A multi-agency protocol for care and investigation	Parents of infants dying of SIDS	Postal questionnaire survey and comments made to support group by other parents	100%
		N = 892		
		United Kingdom		
PR Sexton and SB Stephen [24] 1991	Postpartum mothers’ perceptions of nursing interventions for perinatal grief.	Mothers of infants dying perinatally	Telephone questionnaire survey	50%
		N = 30		
		USA		
DJ Spooren, H Henderick and C Jannes [36] 2000	Survey description of stress of parents bereaved from a child killed in a traffic accident. A retrospective study of a victim support group	Parents of children dying in road traffic accidents	Postal questionnaire survey	25%
		N = 85		
		Belgium		
J Sullivan and P Monagle [32] 2011	Bereaved parents’ perceptions of the autopsy examination of their child	Parents of children undergoing autopsy	Postal questionnaire survey	50%
		N = 53		
		Australia		
B Teklay, LB Wiwe and JL Thomsen [34] 2005	Contact with relatives after forensic autopsies	Relatives of patients having forensic autopsy	Case record review by pathology department	100%
		N = 360		
		Denmark		
F Thuen [37] 1997	Social Support after the Loss of an Infant Child: A Long-Term Perspective.	Parents of infants dying of SIDS	Postal questionnaire survey	Minimal data
		N = 251		
		Norway		
MMT Vennemann, C Rentsch, T Bajanowski and G Zimmer [42] 2006	Are autopsies of help to the parents of SIDS victims? A follow-up on SIDS families.	Parents of infants dying of SIDS	Postal questionnaire survey	100%
		N = 141		
		Germany

**Table 3:** **Details of included qualitative studies**

Authors and year of publication	Name of study	Population and country	Study type	Proportion of study results included
MA Ashby, RJ Kosky, HT Laver and EB Sims [12] 1991	An enquiry into death and dying at the Adelaide Children's Hospital: a useful model?	Parents of children dying in hospital	Interviews with staff and parents, written submissions	Minimal data
		N = 6		
		Australia		
T Bellali, I Papazoglou and D Papadatou [22] 2007	Empirically based recommendations to support parents facing the dilemma of paediatric cadaver organ donation.	Parents who were asked to donate their children’s organs	In-depth interviews with parents	Minimal data
		N = 22		
		Greece		
KL Bright, MB Huff and K Hollon [39] 2009	A broken heart--the physician’s role: bereaved parents’ perceptions of interactions with physicians”.	Bereaved parents, children dying of any age, including adulthood, of any cause	Postal survey with open-ended question	100%
		N = 137		
		USA		
SK Kuhn [27] 2008	The process of parental bereavement following the violent death of a child. PhD Thesis	Parents of children (including young adults) dying in violent deaths	In –depth interviews with parents	Not applicable PhD Thesis
		N = 11		
		USA		
CM Lemmer [13] 1991	Parental perceptions of caring following perinatal bereavement	Parents of infants dying in the neonatal period	In –depth interviews with parents	25%
		N = 28		
		USA		
ME Macdonald, S Liben, FA Carnevale, JE Rennick, SL Wolf, D Meloche and SR Cohen [40] 2005	Parental perspectives on hospital staff members’ acts of kindness and commemoration after a child’s death	Parents of children dying on paediatric intensive care units (PICU)	Field ethnography	50%
		N = 12		
		Canada		
HE McHaffie, IA Laing and DJ Lloyd [29] 2001	Follow up care of bereaved parents after treatment withdrawal from newborns	Parents of infants dying on neonatal intensive care (NICU)	In –depth interviews with parents	100%
		N = 108		
		United Kingdom		
KL Meert, S Eggly, M Pollack, KJS Anand, J Zimmerman, J Carcillo, CJL Newth, JM Dean, DF Willson and C Nicholson [38] 2007	Parents’ perspectives regarding a physician-parent conference after their child’s death in the pediatric intensive care unit	Parents of children dying on paediatric intensive care units (PICU)	In –depth interviews with parents (2007)	100%
		N = 56		
		USA		
KL Meert, S Eggly, M Pollack, KJS Anand, J Zimmerman, J Carcillo, CJL Newth, JM Dean, DF Willson and C Nicholson [46] 2008	Parents’ perspectives on physician-parent communication near the time of a child’s death in the pediatric intensive care unit	Secondary analysis of data from Meert, Eggly et al. [38]		Minimal Data
KL Meert, SH Briller, SM Schim, C Thurston and A Kabel [18] 2009	Examining the needs of bereaved parents in the pediatric intensive care unit: a qualitative study.	Parents of children dying on paediatric intensive care units (PICU)	In –depth interviews and focus groups with parents	75%
		N = 46		
		USA		
EC Meyer, MD Ritholz, JP Burns and RD Truog [15] 2006	Improving the quality of end-of-life care in the pediatric intensive care unit: parents’ priorities and recommendations	Parents of children dying on paediatric intensive care units (PICU)	Open-ended postal questionnaire	Minimal Data
		N = 56		
		USA		
H Nordby and O Nohr [49] 2009	Interactive emergency communication involving persons in crisis	Parents of SIDS infants	Semi-structured interviews with parents	Minimal Data
		N = 11		
		Norway		
EA Pector [30] 2004	How bereaved multiple-birth parents cope with hospitalization, homecoming, disposition for deceased, and attachment to survivors.	Parents of multiple birth infants who die neonatally	Narrative email survey	25%
		N = 70		
		USA		
DE Reilly, JC Huws, RP Hastings and FL Vaughan [14] 2008	‘When your child dies you don’t belong in that world anymore.’ - Experiences of mothers whose child with an intellectual disability has died	Bereaved mothers of children who had an intellectual disability (ID)	In-depth interviews with parents	25%
		N = 9		
		United Kingdom		
AH Schaap, H Wolf, HW Bruinse, S Barkhof-van de Lande and PE Treffers [20] 1997	Long-term impact of perinatal bereavement. Comparison of grief reactions after intrauterine versus neonatal death	Parents of infants dying perinatally	In-depth interviews with parents	Minimal Data
		N = 38		
		The Netherlands		
C Skene [26] 1998	Individualised bereavement care	Parents of infants dying neonatally	Semi-structured interviews with bereaved mothers	Minimal Data
		N = 9		
		United Kingdom		
C Snowdon, DR Elbourne and J Garcia [44] 2004	Perinatal pathology in the context of a clinical trial: attitudes of bereaved parents	Parents of infants dying on neonatal intensive care (NICU)	Semi-structured interviews with bereaved mothers	Minimal Data
		N = 18		
		United Kingdom		
P Swanson, J Brockbank, J Houghton, P Mountbatten, B Read, A Ross and J Woodward [21] 2002	Panel discussion. Grief and bereavement with the loss of a twin	Mothers of multiple birth children dying at any time (including adulthood)	Semi-structured interviews with bereaved mothers	Minimal Data
		N = 66		
		Australia		
S Todd [55] 2007	Silenced grief: living with the death of a child with intellectual disabilities	Bereaved parents of children who had an intellectual disability (ID)	In-depth interviews with parents	Minimal Data
		N = 13		
		United Kingdom		
A Wisten and K Zingmark [16] 2007	Supportive needs of parents confronted with sudden cardiac death--a qualitative study	Parents of children suffering a sudden cardiac death (including adult deaths)	In-depth interviews with parents	100%
		N = 28		
		Sweden

**Table 4:** **Details of included mixed data studies**

Authors and year of publication	Name of study	Population and country	Study type	Proportion of study results included
LK Calhoun [19] 1994	Parents’ perceptions of nursing support following neonatal loss	Parents of infants dying in neonatal units	Questionnaire distributed by support group	100%
		N = 23		
		USA		
SN Covington and SK Theut [28] 1993	Reactions to perinatal loss: a qualitative analysis of the National Maternal and Infant Health Survey	Mothers of infants dying perinatally	Postal questionnaire survey	75%
		N = 413		
		USA		
A Dent [47] 2000	Support for families whose child dies suddenly from accident or illness. PhD Thesis	Parents of children dying suddenly	Postal questionnaire survey	Not applicable – PhD thesis
		N = 72		
		United Kingdom		
K Dyregrov [54] 2002	Assistance from local authorities versus survivors’ needs for support after suicide	Parents of children who committed suicide	Postal questionnaire survey with in-depth interviews for a sample of participants	50%
		N = 128		
		Norway		
HE McHaffie, PW Fowlie, R Hume, IA Laing, DJ Lloyd and AJ Lyon [41] 2001	Consent to autopsy for neonates	Parents of infants dying on neonatal units	In-depth interviews with parents	75%
		N = 108		
		United Kingdom		
E Merlevede, D Spooren, H Henderick, G Portzky, W Buylaert, C Jannes, P Calle, M Van Staey, C De Rock, L Smeesters, et al. [17] 2004	Perceptions, needs and mourning reactions of bereaved relatives confronted with a sudden unexpected death	Relatives of people dying suddenly	Structured interview and analysis of clinical records	25%
		N = 74		
		Belgium		
L Sterry and L Bathgate [35] 2011	Scottish Cot Death Trust Project Report	Parents of infants dying of SIDS	Internet or postal questionnaire survey	75%
		N = 109		
		United Kingdom		

### Results of critical appraisal

18 studies recruited directly from bereavement support groups which parents had to actively choose to join; thus these parents’ experiences may be different from those choosing not to join. The quantitative studies recruitment rate varied from 22 to 100%; in 7 studies the recruitment rate could not be calculated as it was unclear how many eligible families had been contacted.

Death in childhood is associated with lower socio-economic status [11] and this should be reflected in the socio-economic status of participating parents; however only 28 studies provided these data. Reporting studies only gave brief details describing ‘most’ participating parents as white (75-100%), married (70-100%), completing some higher education (50% university, mean of 13–14 years in education) and earning higher than average incomes.

It was difficult to judge the reliability of results in 10 studies due to limited details of data collection, development of questionnaires or interview schedules and methods of qualitative analysis. Despite these deficiencies no studies were excluded; this was to ensure no parental experiences are lost but where necessary these issues are highlighted along with the results of these studies.

The results of the critical appraisal process are given in Additional files 1 and 2.

### Narrative synthesis of results

**Figure 2:**
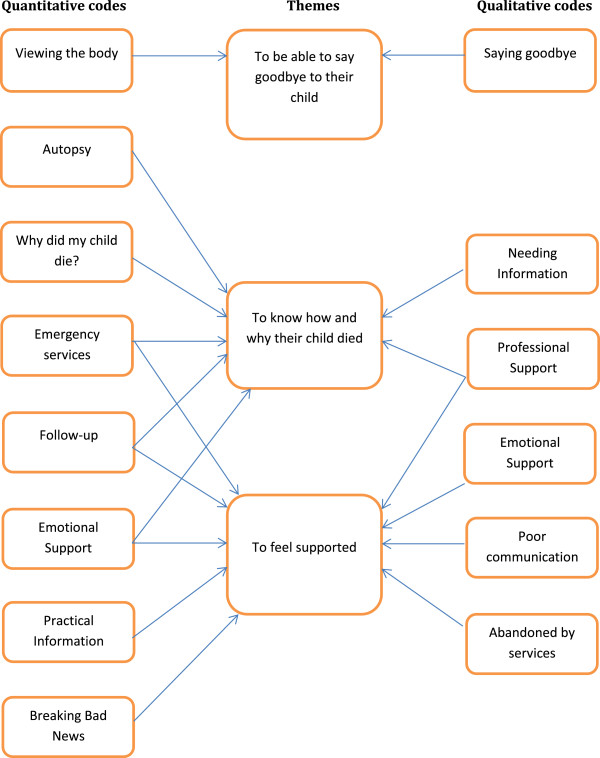
**Quantitative codes, qualitative codes and themes.**

**Figure 3:**
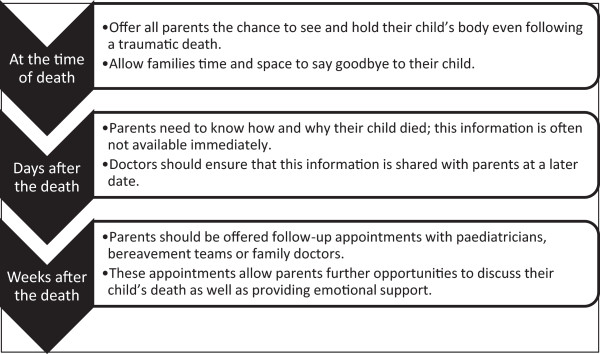
**Summary of recommendations for health care professionals.**

Three themes emerged from the review on what bereaved parents want from professionals after the death of their child: to be able to say goodbye, to understand why and how their child died, and to feel supported by professionals.The themes are shown in Figure 2. A summary chart for health care professionals is shown in Figure 3.

#### Parents want to be able to say goodbye to their child

In the reference framework parents wanted time to hold or be with their children after death, to say goodbye even if the body was mutilated; parents deeply regretted this if they were unable to do so [9, 10]. These were consistent findings across many studies of all types of child death; with qualitative studies detailing parents’ desire for privacy, a peaceful space and adequate time to be able to say farewell [12–20].

In interview studies, parents have described seeing or holding their infant or child’s body as helpful and that not being able to do so increased their grief [16, 21–23]; however survey findings of this are less certain. In one study after perinatal death 30/30 mothers found seeing the baby helpful [24] whereas only 6/21 parents found this helpful in a study of a wider range of child deaths [25]. Parents may choose not to see their child after death, but one-third of parents in a large qualitative study (n = 38) expressed regret that they decided not to see their baby after a perinatal death [20]. However, even when warned of potential regret, a minority of neonatally bereaved parents still felt strongly that they did not want to see their baby [26].

Qualitative studies have reported that parents may still wish to see their child after a traumatic death although others may prefer to remember them unhurt [27]. When parents do not see their child they often imagine the injuries to be worse than they really are [17].

#### Parents want to know how and why their child died

Many different studies of all types of child death confirmed the parental need for information about their children’s deaths identified in the reference framework [17, 22, 28–33]. Conversely, a case record review showed that only 28% of relatives sought results of forensic autopsy examination despite these not being available from any other source; families may not though have been aware that they could seek these results [34].

Both surveys and interview studies have reported that information after any type of child death may help parents make sense of the death and help with their grief [16, 27, 28, 35–37]. Interview studies reveal that information about the death reassures parents that children did not suffer and everything possible was done to save them [16, 17, 29]. Similarly, detailed information reassures parents that their actions were appropriate, helping to diminish some of their feelings of guilt [15, 17, 35, 38] *.*

Parents want to know the cause of death especially for sudden unexpected deaths; the lack of explanation for SIDS may result in further parental distress [10]. Consistent with this a survey of 892 SIDS parents found that finding the cause for death was of the greatest importance for parents [31] and a survey of 413 perinatally bereaved mothers showed that 21% were struggling to understand why their baby died with 51% wanting further information [28].

A consistent finding of studies of all types of child death is that parents have requested follow-up appointments with professionals to ask for further information as at the time of the death they were too distressed to comprehend detailed answers [16, 17, 28, 38, 39]. However, parents have commented in interview studies that returning to the hospital may cause distress from traumatic memories [29, 40], and that following a neonatal death there may be an additional burden of appointments in several different departments [29].

Bereaved parents have described, in interviews, how the lack of information has led them to assume that it is being deliberately withheld [9, 28] and following violent deaths their determination to obtain information from the authorities [27].

##### Autopsy

An interview study of neonatal deaths found that autopsy results may be a powerful tool in helping parents reach a sense of closure [41]; similar results were shown in a survey of SIDS parents where 66% (93/141) believed that mandatory autopsy had helped resolve their grief, even for the 17% (24/141) parents who had not wanted the autopsy initially [42]. Conversely with autopsy of older children, a survey showed only 40% of parents found the results useful and 38% thought the results helped with their grief; however this survey had a low response rates so these results may not be generalizable [32].

Interview studies and surveys have detailed parents’ reasons for consenting to autopsies: to obtain further information about neonatal deaths and future pregnancies in particular was the reason given by ‘the majority of parents’ [41] and by 50% of parents in another neonatal study [43]. Bereaved parents following all types of child death wanted information from their child’s autopsy to help other families in the future [32, 41, 44]. Around half of parents who declined neonatal autopsy in two studies did so because they had no unanswered questions and half because they do not want their baby’s body traumatised further [41, 43].

Surveys and interview studies have shown that a small minority of parents, after consenting to child autopsy subsequently regret it, this ranges from 6-8% [32, 43, 45]; but after refusing a neonatal autopsy some parents regret the loss of potential information, this ranges from 7% [43] of those declining autopsy to 30% [45]. Thorough explanations of the autopsy process are needed, particularly if parents are going to view their children again afterwards, sanitising explanations prior to autopsy may result in more distress later [44].

In Dent et al., some parents struggled to understand the autopsy results despite explanations from professionals [10]. Consistent with this finding other studies have shown parents not receiving autopsy results despite giving consent to the procedure; this happened in 4/13 intensive care deaths [23]. After sudden cardiac death some parents received autopsy results by post so lacked the opportunity to discuss the results with a clinician [16] and a study of paediatric autopsy reported that only 42/52 parents had results explained to them [32]. Parents have reported not understanding explanations of results and thus feeling that their questions remained unanswered [28, 35]; this was the case for 8/16 mothers following neonatal autopsy [45] but in a much larger survey of neonatal autopsy 101/120 parents thought the results were explained appropriately and only 16/120 parents wanted further explanation [43].

#### Parents want to feel supported by professionals

##### Emotional support

Parents felt supported by professionals who showed they were upset when breaking bad news; conversely they were offended if professionals were cold and unemotional. Many parents felt uncared for by the hospital immediately after their child’s death often being left to arrange their own way home [9].

Consistent with the reference framework, other studies of all child deaths report that parents appreciate staff members showing emotion [15, 19, 27, 30, 46–48] and mothers interviewed after a neonatal death interpreted staff who lacked emotion as being uncaring [13]. Similarly other surveys reported on a lack of care shown to parents; 20% (83/413) of perinatally bereaved parents commented on a lack of sensitivity and care by their caregivers [28] and 37/70 parents were dissatisfied with hospital staff after road traffic accident deaths [36].

Other studies have given further details of parents’ experiences of emotional support; doctors are valued as guiding parents through the crisis of their children’s deaths [18, 39], social workers and chaplains have been important to parents after intensive care deaths [23], police officers have been supportive with sudden deaths but their presence can be upsetting for some due to the implication that a crime may have occurred [16, 35]. Parents may clearly remember interactions with professionals at the time of their children’s deaths; later these memories may bring comfort or distress for both hospital [18] and community deaths [49].

Most parents wanted mementoes of their child but these were offered to less than half of families [10]. Other studies confirm the significance of these mementoes or returning a child’s clothing and possessions after sudden deaths [33, 50] or those on intensive care [23]; photographs may be particularly valued after a perinatal death [24].

##### Emergency services

In the reference framework there were mixed findings with some but not all parents praising the police for their support [9]. In Dent et al., most parents were happy with the emergency services although 28% of parents thought the police unsympathetic and one-third of parents were not allowed to accompany their child in the ambulance [10].

Only 4 studies, all of SIDS, detailed parents’ views of the police; these were similarly mixed. In one study 48% of parents thought the police were kind and helpful, but 30% felt they were unhelpful and treated parents as guilty and assumed that a crime had been committed [35]; another study commented on disproportionate police involvement [51]. Conversely, in Ireland, satisfaction with police services following SIDS is high with 86/100 parents finding police helpful [52] and 75% of 69 parents stating that police carried out the process of identification sensitively [53]. These results are surprising given that there is a similar level of involvement by UK and Irish police in SIDS cases.

Only two other studies reported parents’ views on ambulance services. 50% of 109 SIDS parents thought ambulance staff were helpful but 21% criticised ambulance staff for seeming to panic and being ill-equipped to deal with infants [35]. 41/80 parents were dissatisfied with ambulance services following road traffic accident deaths [36].

##### Professional support

In Finlay and Dallimore, the most helpful support for parents was on-going contact with a professional present at the time of death [9]. In Dent et al., parents wanted more practical information about dealing with the bereavement and for professionals to remain in contact with them [10]. As in the reference framework, bereaved parents in other studies wanted continuing contact with medical teams both after sudden deaths and those in hospital [18, 35, 38, 47]. This is particularly important after sudden deaths and suicides, as grief-stricken parents may feel unable to contact professionals themselves, suggesting contact should be offered routinely and continued for some months [16, 47, 54]. In interview studies parents have explained that they want professionals to show that they care about them and their family after the death [29, 38, 39], sharing memories of the child is an important part of this [29, 39] as is attending funerals or offering formal condolences [18, 30, 35, 39, 40].

Parents in interview studies described feeling abandoned by professionals when contact stops after a child death having grown close to staff during prolonged hospital stays [12, 18, 38] or with the abrupt cessation of support services after deaths of children with intellectual disability [14, 55]. Similar feelings of abandonment by professionals are also felt by parents after sudden cardiac death [16] or SIDS [35] despite their families not being known to services prior to the death.

##### Follow-up of bereaved parents by physicians or other health professionals

In Finlay and Dallimore, only 16/120 families had any hospital follow-up [9]. In Dent et al., more than half of parents had no follow-up a with a hospital paediatrician; of those who did 88% found it helpful. Very few families had formal follow-up with the GP or health visitor but all of these found this helpful [10].

Rates of hospital follow-up for bereaved parents were very variable ranging from 16% to 77% for SIDS [50, 52], 77% for deaths on paediatric intensive care [56] and 92% for neonatal deaths [29]. Similar to the reference papers, in 13 studies parents stated that they would like more medical follow-up after all child deaths [14, 17, 18, 27, 29, 31, 38, 47, 50, 57–60] and no study reported parents wanting less contact with professionals.

Although Dent et al. reported high rates of parental satisfaction with paediatric follow-up [10] lower rates were found in other studies: 56% and 63% for SIDS [35, 58], 33% for perinatal deaths [25] and 62% for pediatric intensive care deaths [56]. Again, unlike in Dent et al., in one study only half of parents were satisfied with GP or health visitor follow-up after SIDS [35] although other parents have commented that they found comfort by talking to their health visitor as she had known the child in life [47]. Surveys of bereaved parents showed that parents appreciated follow-up appointments where paediatricians have explained the cause for infant deaths [57, 58] and offered emotional support in the longer term [57, 61]. For SIDS parents, such emotional support from professionals is associated with increased positive affect up to 5 years after the death [37]; however, not all parents will want emotional support [42].

##### Good communication

In Finlay and Dallimore, twice as many parents said that the bad news had been broken in a sympathetic manner compared to those who did not [9]. In Dent et al., all parents reported that they had been told sensitively about their child's death [10].

Other surveys show varying rates of satisfaction with breaking bad news, from 46% following sudden deaths at any age to 62% following child deaths in road traffic accidents [36] and 87% after neonatal deaths [59]. Dissatisfaction after any sudden death in children or adults was mainly associated with a lack of information [17].

Other qualitative studies give further details from a wide range of child deaths. When breaking bad news professionals’ language should be appropriate for the parents’ to understand, not give false hope but not be so factual as to give offence; parents should be given time to assimilate information prior to addressing other issues [39, 46]. Parents want to feel listened to at the time of the death [14, 28] and subsequently [21, 27]. Parents have reported that sometimes professionals lack compassion [39], dismiss their feelings [27], avoid parents [30], or show them outright hostility [27] and openly judge their lifestyles or parenting choices as their children lie dying [18]. Parents have also described actions by professionals that are inappropriate and insensitive: handing bereaved mothers routine well-baby information [13], suggesting infant deaths are ‘God’s Will’ or that mothers can have another baby; and suggesting that parents should be satisfied as they have surviving infants in deaths following multiple pregnancies [21, 30].

## Discussion

Our review found that parents wish to be able to say goodbye to their child at the hospital; staff need to ensure that families are welcomed and that they are given time and privacy to say their farewells. Receiving timely and appropriate information about their child’s death is an important part of the grieving process for parents. Parents value emotional support from professionals at the time of death and in the subsequent weeks and months. Parents appreciate follow-up appointments with professionals both to help them understand why their child died and as a way of offering continuing support to the family. These findings can be used by any professional supporting bereaved parents within health care, police or social services.

This review includes the experiences of over 4000 bereaved parents whose children died unexpectedly at any age. The review has highlighted considerable consistency in what parents report as their needs following an unexpected child death, despite differing ages of children and causes for deaths. There was a significant overlap in findings in many studies and theoretical saturation was reached before all papers were coded; thus it is unlikely that any significant themes have been missed. The review is however limited by the lack of papers published on interactions with police or other agencies so the findings may have limited applicability outside of health care. Most of the studies recruited mainly from white families with above average incomes; this may reduce the generalizability of the results as child deaths occur more commonly with social deprivation.

This literature review includes data on child deaths of all ages; the only comparable similar systematic reviews are of parents’ experiences of perinatal deaths. These results are similar in that parents found holding their baby after death to be beneficial and wanted more information on why their baby died [62].

## Conclusions

It is clear from this literature review that parents would like to be offered more support from professionals after child death; the support should not finish when parents leave the hospital without their child. Hospital staff should be trained to support parents at the time of child death and policies put in place to ensure families are able to say goodbye to their child in a dignified way. Clinical staff should ensure that contact is maintained with bereaved parents and they are invited back for follow-up appointments to discuss their child’s death as a matter of routine; no parent should be left with unanswered questions about their child’s death.

As child death review (CDR) processes become more elaborate there remains the potential for this to become an intrusive process for the parents; although parents may obtain more information as to the cause of death, the enquiry process may increase their distress. CDR is now undertaken in many countries so the potential for parental distress is great. It is essential that all such processes are developed and delivered in ways that are supportive to parents, help them to understand the reasons why their child died, and enable them to say goodbye to their child in an appropriate and supported way.

## Authors information

JG is currently evaluating multi-agency working following Sudden Unexpected Death in Infancy. PS is a Designated Doctor for unexpected child deaths and has published extensively on the investigation of unexpected child deaths and SIDS.

## Electronic supplementary material

Additional file 1: Table S1: Critical Appraisal of quantitative studies. (DOCX 46 KB)

Additional file 2: Table S2: Critical Appraisal of qualitative studies. (DOCX 44 KB)

## Abbreviations

SIDS: Sudden infant death syndrome
CDR: Child death review.
