# Supplementary material for: What do bereaved parents want from professionals after the sudden death of their child: a systematic review of the literature
Source: BMC Pediatr. 2014 Oct 15;14:269. doi: 10.1186/1471-2431-14-269 (PMC4287432; doi:10.1186/1471-2431-14-269)
Supplement: Supplementary file 1 — Additional file 1: Table S1: Critical Appraisal of quantitative studies. (DOCX 46 KB) [file 12887_2014_1193_MOESM1_ESM.docx]

Table S1 Critical Appraisal of quantitative studies

| Study | Statement of aims | Survey type | Development of survey questions | Piloting and Validation of survey | Sampling Frame and Design | Response rate | Efforts to improve response | Details of Socio-economic status of participants |
| --- | --- | --- | --- | --- | --- | --- | --- | --- |
| [Ahrens, Hart et al. (1997](#_ENREF_1)) | To identify healthcare provider actions that will facilitate bereaved families recovery | Postal questionnaire survey | Unknown method | Questionnaire reviewed by SIDS parents support group | Parents contacted from SIDS support group mailing list - risk of inherent bias, deaths up to 16 years prior to survey | 62%  37/60 parents completed survey | no | None given |
| [Calhoun (1994](#_ENREF_5)) | To confirm appropriateness of nursing interventions after death of infant | Questionnaire distributed by support group | Guided by previous research study | Questionnaire reviewed by expert panel and piloted with bereaved parents | Parents attending a parental loss support groups, risk of inherent bias as participants selected by support group co-ordinator | 55%  23 complete surveys and 42 partially complete surveys included | no | Mean age 31 years, all parents married |
| [Dent, Condon et al. (1996](#_ENREF_8)) | To establish parents views on care received after the sudden death of their child | Postal questionnaire survey | Advised by bereaved parents | Pilot study completed | Parents of any child dying suddenly in study area, risk of bias as GP needed to agree to family to be contacted, access denied in several cases | 58%  GP agreed access to 72/185 eligible families,  42/72 families completed survey | no | None given |
| [Dent (2000](#_ENREF_7)) | To see if a bereavement assessment tool helps HV to work with bereaved parents | Postal questionnaire survey | Advised by bereaved parents and experience Health Visitors | Pilot study completed | Parents of any child dying suddenly in study area notified by emergency departments or ambulance control. | 59%  72/122 completed survey | 1 reminder letter sent | Most parents married, all white british ethnicity |
| [DiMarco, Menke et al. (2001](#_ENREF_9)) | To determine if attending a support group helps after perinatal loss | Postal questionnaire survey | Standardised tool used | Previously validated | Parents on mailing list of perinatal loss newsletter, some of whom attended support groups  (not clear if parents had to opt in for newsletter) | 32%  128/400 parents completed survey | 2 reminder letters sent | Most participants white and married |
| ([Dyregrov 2002](#_ENREF_10)) | To describe what help suicide survivors want and receive | Postal questionnaire survey with interviews for a sample of participants | Unknown method for bespoke questionnaire, also standardised tool | Unknown for bespoke questionnaire  Previously validated tool Kvale method for interviews and analysis | Parents of all suicide cases in 18 month period details obtained from police records | 77%  128/166 parents completed survey | No | Participants similar to non-participants but no further details. |
| [Finlay and Dallimore (1991](#_ENREF_11)) | To determine parents views on how the death of their child should have been handled | Postal questionnaire survey | Unknown method | Unknown | Parents who were members of bereavement support organisation, risk of inherent bias as only support group members participating | 80%  120/150 families completed survey | no | None given |
| [Harper and Wisian (1994](#_ENREF_12)) | To determine which physician behaviours are helpful to bereaved parents | Postal questionnaire survey or questionnaire distributed at support group | Unknown method for bespoke questionnaire, also standardised tool | Unknown for bespoke questionnaire  Previously validated tool | Parents referred to perinatal loss support group by hospital regardless of whether they were attendees or not | Unknown  37 families completed survey from unknown total | no | Mean age 30. Middle-lower income |
| [Hazzard, Weston et al. (1992](#_ENREF_13)) | To determine which experiences of bereaved parents affect grief | Structured interview | Bespoke questionnaire similar to one published previously. Standardised tool also used | Unknown for bespoke questionnaire  Previously validated tool | Parents of children dying in hospital in study period and from bereavement support group. Risk of bias as permission needed from doctors to contact parents only given in 88% of cases, and support group recruitment. Analysis showed non-responders similar to responders | Unknown 18/59 families from hospital and additional 16 parents from support group completed survey | Bereave-ment support group used to increase recruit-ment | 51% parents >35 years, 65% earned > $30K per annum |
| [Krauel Vidal, Silva Vazquez et al. (1992](#_ENREF_14)) | To examine the care of parents when a baby dies on NNU | Postal questionnaire survey | Unknown method | Unknown | Parents of babies dying on NNU – no further details given | 27%  49/180 families completed survey | no | None given |
| [Laakso and Paunonen-Ilmonen (2002](#_ENREF_16)) | To find tools to help healthcare workers support bereaved mothers | Questionnaire and structured interview | Unknown method for bespoke questionnaire, also standardised tool | Unknown for bespoke questionnaire  Previously validated tool | Parents of children dying in 4 year time period – no further details given | 54%  91/169 parents  completed survey | no | None given |
| [Livesey (2005](#_ENREF_18)) | To see how a multi -agency SUDI protocol works in practice | Postal questionnaire survey as part of audit of practice | Unknown method | Unknown | Parents of all SUDI cases in study area in set time period | Unknown | no | None given |
| [Macnab, Northway et al. (2003](#_ENREF_20)) | To determine which staff behaviours are helpful to parents of children dying on PICU | Questionnaire and structured interview | Unknown method for bespoke questionnaire, also standardised tool | Unknown for bespoke questionnaire  Previously validated tool | Parents of all local children dying on PICU in set time period, risk of bias as non-local families and coroners cases excluded | 100%  13/13 families  completed survey | Not needed | All approached participated but more than50% had completed higher education |
| [McDonnell, Cullen et al. (1999](#_ENREF_21)) | To evaluate parents’ experiences of services by professionals after SIDS | Structured interview | Unknown method | Unknown | All parents from a national SIDS register | 70%  131/188  families  completed survey | no | None given |
| [Merlevede, Spooren et al. (2004](#_ENREF_27)) | To identify the needs of suddenly bereaved relatives | Structured interview and analysis of clinical records | Unknown method for bespoke questionnaire, also standardised tool | Unknown for bespoke questionnaire  Previously validated tool | Relatives of all sudden death cases approached identified by hospital records. Non responders were relatives of older adult dead patients | 48%  53/110 relatives completed interview | 1 telephone call reminder | None given |
| [Meyer, Burns et al. (2002](#_ENREF_28)) | To examine priorities of parents at time of child death on PICU | Postal questionnaire survey | Bespoke questionnaire based on clinical experience and literature review | Questionnaire reviewed by professionals and piloted with bereaved parents | Parents of all children dying on a PICU in 2 year time period | 58%  56/96 families  completed survey | no | Mean age 42, 75% married 91% Caucasian |
| [Neidig and Dalgas-Pelish (1991](#_ENREF_30)) | To collect information from bereaved parents regarding health professionals’ interventions | Postal questionnaire survey | Unknown method for bespoke questionnaire, also standardised tool | Unknown for bespoke questionnaire  Previously validated tool | Convenience sample of parents from bereavement support group, risk of bias due to this, wide time frame of bereavement from months to many years | Unknown  22 parents  completed survey from unknown total | no | All white, all completed high school, 57% completed higher education |
| [Oliver, Sturtevant et al. (2001](#_ENREF_32)) | To determine which initiatives helped bereaved parents | Structured interview | Unknown method | Unknown | All parents completing a hospital bereavement program, risk of bias dropouts not recruited | 70%  54/77  families  completed survey | No | None given |
| [Ostfeld, Ryan et al. (1993](#_ENREF_33)) | To better understand characteristics and resources that effect the grieving process after SIDS | Postal questionnaire survey | Questionnaire based on similar SIDS one used by authors previously | Questionnaire based on similar SIDS one used by authors previously | Parents from a statewide SIDS support group, whose baby died in set time period, risk of bias from support group recruitment | 44%  38/86 families completed survey | no | Responders older than non responders |
| [Powell (1991](#_ENREF_35)) | To assess if the presence or absence of social support influences outcome for SIDS parents | Structured interview | Unknown method for bespoke questionnaire, also standardised tool | Unknown for bespoke questionnaire  Previously validated tool | Parents of all SIDS cases in one region, data from national register. | 66%  40/61 families completed survey | no | None given |
| [Rahman and Khong (1995](#_ENREF_36)) | To learn of bereaved parents views on perinatal autopsy | Telephone questionnaire survey | Unknown method | Unknown | Mothers of infants dying in perinatal period; details from a hospital list. | Unknown  29 mothers completed survey | no | None given |
| [Rankin, Wright et al. (2002](#_ENREF_37)) | To describe bereaved mothers experience and views of infant autopsy | Postal questionnaire survey | Unknown method | Unknown | Mothers attending a hospital bereavement service – inherent risk of bias | 63%  166/258 mothers completed survey | 2 reminders sent | Mean age 32 |
| [RCPath and RCPCH (2004](#_ENREF_38)) | To seek parents views on SIDS national support group campaign for improved SUDI investigation | Postal questionnaire survey and comments made to support group by other parents | Unknown method | Unknown | Parents on mailing list of support group and all comments made to national support group by parents in one year. Inherent risk of bias but huge survey | 28%  893/3200 families completed survey and further 1046 other contacts from parents | no | None given |
| [Sexton and Stephen (1991](#_ENREF_41)) | To determine maternal perceptions of nursing interventions for bereavement support | Telephone questionnaire survey | Questionnaire development guided by literature review | Unknown | Mothers attending perinatal bereavement support programme– inherent risk of bias | 54%  30/56 mothers completed survey | no | 23% of mothers 23 years or younger |
| [Spooren, Henderick et al. (2000](#_ENREF_44)) | To assess parents views on support received at time of child's death from RTA | Postal questionnaire survey | Unknown method for bespoke questionnaire, also standardised tool | Unknown for bespoke questionnaire  Previously validated tool | All parents on mailing list of national support group for families of children killed in RTA. Risk of bias from mailing list recruitment. Deaths of adult children included. | 41%  55/133 families completed survey | no | None given |
| [Sullivan and Monagle (2011](#_ENREF_46)) | To explore bereaved parents’ views on autopsy | Postal questionnaire survey | Questionnaire development guided by literature review and clinical practice, also standardised tool | Bespoke questionnaire reviewed by bereaved parents.  Previously validated tool | Parents on mailing list of bereavement support newsletter who then requested to receive the questionnaire about experience of autopsy. Risk of bias from mailing list recruitment. | Unknown  53 families completed survey.  831 families on mailing list | no | None given |
| [Sterry and Bathgate (2011](#_ENREF_45)) | To report bereaved families experiences following SUDI | Internet or postal questionnaire survey | Unknown method | Unknown | All parents on mailing list of national support group for SIDS – inherent risk of bias. Wide time frame of bereavement from months to many years | 22%  109/487 families  Completed survey | no | None given |
| [Teklay, Wiwe et al. (2005](#_ENREF_48)) | To see how often relatives seek the results of forensic autopsy | Case record review by pathology department | Not applicable | Not applicable | All forensic autopsy cases in one year in department. Includes adult deaths. | All 380 cases included | Not applicable | Not applicable |
| [Thuen (1997](#_ENREF_49)) | To assess the relationship between support and long-term psychological adaption of bereaved SIDS parents | Postal questionnaire survey | Unknown method for bespoke questionnaire, also standardised tool | Unknown for bespoke questionnaire  Previously validated tool | Parents on mailing list of national SIDS support group. Risk of bias from mailing list recruitment. Wide time frame of bereavement from months to many years | 27%  169/630 families competed survey | no | 86% of parent still married, mean education duration 13 years |
| [Vennemann, Rentsch et al. (2006](#_ENREF_51)) | To assess bereaved parents views about autopsy following SIDS | Postal questionnaire survey | Unknown method | Unknown | Parents recruited from those taking part in previous national SIDS study. | 38%  141/373 families completed survey | 1 reminder sent | Participants were of higher SES than non-participants, but otherwise similar |
