# Supplementary material for: What do bereaved parents want from professionals after the sudden death of their child: a systematic review of the literature
Source: BMC Pediatr. 2014 Oct 15;14:269. doi: 10.1186/1471-2431-14-269 (PMC4287432; doi:10.1186/1471-2431-14-269)
Supplement: Supplementary file 2 — Additional file 2: Table S2: Critical Appraisal of qualitative studies. (DOCX 44 KB) [file 12887_2014_1193_MOESM2_ESM.docx]

Table S2 Critical Appraisal of qualitative studies

| Study | Statement of research aims | Appropriate research design | Recruitment strategy appropriate | Does data collection address research issue | Relationship between researcher and participants | Ethical Issues | Rigorous data analysis | Details of Socio-economic status of participants |
| --- | --- | --- | --- | --- | --- | --- | --- | --- |
| [Ashby, Kosky et al. (1991](#_ENREF_2)) | To enquire into the management of children who are dying and their families | Interviews with staff and parents, written submissions | Not stated how families recruited | No details given | Not stated who carried out interviews | Not stated | No qualitative analysis undertaken, results purely descriptive | None given |
| [Bellali, Papazoglou et al. (2007](#_ENREF_3)) | To identify needs and expectations of parents from health care professionals around the time of child organ donations | In-depth interviews with parents | Families identified through medical records of children dying as potential organ donors | In-depth interviews at parental home, recorded and transcribed | Interviews piloted  No description of interviewer | Mental health follow-up arranged for parents if needed, ethical guidelines followed | Detailed description given, analysis by 3 researchers | None given |
| [Bright, Huff et al. (2009](#_ENREF_4)) | To enhance understanding of how bereaved parents feel about interactions with physicians at the time of child death | Open ended questions at end of postal survey | All bereaved parents on statewide mailing list approached | Not stated | No face to face contact | Ethical approval obtained for study | Nvivo software used no further details given | 45% of parents were college grads, 97% were white |
| [Covington and Theut (1993](#_ENREF_6)) | To analyse the answers of bereaved mothers to an open-ended question | Open-ended question on national maternal & infant health survey | Survey sent to >8000 bereaved mothers, 400 answered open-ended question | Not stated but very wide question used | No face to face contact | Not stated | 2 authors analysed data, no further details given | None given |
| [Kuhn (2008](#_ENREF_15)) | What is bereavement process of parents following violent death of a child? | In-depth interview with parents | Parents contacted via victims crime unit office | In depth interviews at parental home or location of their choice. Interviews recorded and transcribed. Data saturation reached | Discussed – little detail given | Ethical approval obtained for study. Counsellor available for participants if needed | Grounded theory used, solo analysis (PhD thesis) | 7/11 parents completed high school, all were white |
| [Lemmer (1991](#_ENREF_17)) | What do perinatally bereaved parents perceive as care-giving from nurses or physicians? | In –depth interviews with parents | Parents contacted via hospital or bereavement support groups | In depth interviews at parental home or location of their choice. Interviews recorded and transcribed | Interviews piloted  No description of interviewer | Ethical approval obtained for study. | Grounded theory used, 2 authors analysed data, full details of analysis given | Parents had higher than average income, mean of 14 years education for mothers, 15 years for fathers |
| [Macdonald, Liben et al. (2005](#_ENREF_19)) | To understand experiences of parents whose children die on PICU | Field ethnography method | Parents contacted via hospital list of deaths | In depth interviews at parents’ home or location of their choice. Interviews recorded and transcribed, extensive field notes used | Details of interviewers given, relationship not further discussed | Ethical approval obtained for study | Whole team worked on analysis, full details given | Multicultural sample of parents |
| [McHaffie, Fowlie et al. (2001](#_ENREF_22)) | To determine parents views on autopsy after withdrawal of treatment on NNU | In –depth interviews with parents | Parents recruited from neonatal follow-up interview | Location of interview not stated. Interviews recorded and transcribed | Not stated | Ethical approval obtained for study | Sample of data coding checked for consistency with other authors. No further details of analysis | More teenagers and unemployment in non -participants than participants but still reflective of neonatal unit population |
| [McHaffie, Laing et al. (2001](#_ENREF_23)) | To explore parents perceptions of bereavement care following a death on NNU | Secondary analysis of data from McHaffie, Fowlie et al. (2001 | | | | | | |
| [Meert, Eggly et al. (2007](#_ENREF_25)) | To investigate parents perceptions of desirability of a conference with the physician after child death on PICU | In-depth interviews with parents | All parents of children dying on PICU in preceding 12 months approached | Telephone interview, recorded and transcribed. Interview guide led by literature review, piloted. Data saturation reached | Research assistants conducted interviews, quality control by authors | Ethical approval obtained for study | Analysed by 2 authors, doctor and behavioural scientist independently, 3^rd^ person checked sample for consistency. Bereaved parents reviewed analysis. Software used, thematic analysis, Full details given. | 75% of parents were white, 52% were college graduates, 70% were married, employed or homemaker |
| [Meert, Eggly et al. (2008](#_ENREF_26)) | To describe parents perspectives on physician communication at time of child death on PICU | Secondary analysis of data from Meert, Eggly et al. (2007) | | | | | | |
| [Meert, Briller et al. (2009](#_ENREF_24)) | To gain a greater understanding of parents needs around the time of child death on PICU | In-depth interviews with parents, focus groups with bereaved parents | All parents of children dying on PICU in 6 month period approached | Interview guide based on previous research and literature. Interviews and focus groups in hospital, videotaped and transcribed | Interview by PICU physician and chaplain jointly, focus group lead by medical anthro-pologist | Ethical approval obtained for study | Thematic analysis, full research team involved in process, full details given. | Individual parents 55:45 white: black, focus group 85:15  white: black |
| [Meyer, Ritholz et al. (2006](#_ENREF_29)) | To identify parents priorities for end of life care | Open ended questionnaire sent to bereaved parents | All parents on children dying on PICU in 2 year period | Questionnaire based on clinical experience and literature review. Piloted | No face to face contact | Ethical approval obtained for study | No analysis of open –ended answers quotes given verbatim in report | 75% were married, 91% were white |
| [Nordby and Nohr (2009](#_ENREF_31)) | To understand how parents experience communication, care and empathy with emergency telephone operators | Semi-structured interviews | Bereaved parents recruited via national SIDS support group – random selection but 6 urban 5 rural | Discussion of methodology and theoretical perspectives but no further detail given | Not stated | Not stated | Not stated | Mix of urban and rural cases |
| [Pector (2004](#_ENREF_34)) | To elicit bereaved multiple birth parents perceptions regarding support | Narrative email survey | Bereaved parents recruited via support groups and websites | No details of how survey developed | No face to face contact. | Ethical approval not required for internet survey. | Eaves grounded theory approach used. Transcription checked with participants. Single author analysis | Most parents were married |
| [Reilly, Huws et al. (2008](#_ENREF_39)) | To learn of the experiences of bereaved parents whose child had an intellectual disability (ID) | In-depth interviews | Bereaved parents recruited from support groups, charities and hospices. Homogenous sample from bigger study | Interview schedule developed from published questionnaire, piloted | Interviewer was research student with experience of ID, discussed in detail | Ethical approval obtained for study | Interpretive Phenomenological Analysis used, 2 researchers analysed results | None given |
| [Schaap, Wolf et al. (1997](#_ENREF_40)) | To describe long term effects after perinatal death | In-depth interviews with parents | Bereaved parents who had taken part in previous perinatal study contacted via GP | No details of how interview schedule developed, interview recorded and transcribed | Not stated | Ethical approval obtained for study | No details given | None given |
| [Skene (1998](#_ENREF_42)) | To hear individual stories of bereaved mothers | Semi-structured interviews with bereaved mothers | Bereaved mothers from one regional neonatal unit | Interview schedule guided by literature review, piloted. Interviews recorded and transcribed | Role of interviewer discussed | Ethical approval obtained for study | Coded and themes described by single author. No further details given. | None given |
| [Snowdon, Elbourne et al. (2004](#_ENREF_43)) | To report attitudes of neonatally bereaved mothers to autopsy | Semi-structured interviews with bereaved mothers | Bereaved parents of neonates who had been part of medical trial. Access to parents via neo-natologists (with some difficulties) | Interview schedule guided by literature review. Interviews recorded and transcribed | Not stated | Ethical approval obtained for study | Analysed by identifying and grouping themes by research team, computer software used. No further details given | None given |
| [Swanson, Brockbank et al. (2002](#_ENREF_47)) | To study nature of bereavement in mothers of dead multiple birth child | Semi-structured interviews with bereaved mothers | Mothers recruited via multiple birth child health study and bereavement groups | Interview schedule developed from literature and refined after first 10 interviews. Interviews recorded and transcribed. Location not given. | Not stated | Ethical approval obtained for study | No details of qualitative analysis given. Results checked with bereaved mothers in focus groups | All parents were white and English speaking with wide range of socioeconomic status |
| [Todd (2007](#_ENREF_50)) | To examine the bereavement experiences of parents of children with intellectual disabilities (ID) | In-depth interviews with bereaved parents of children with ID | Recruited via newsletter and personal contacts (support groups for ID unsuccessful) | Not stated how interview schedule developed. Interview in parents’ home Interviews recorded and transcribed. | Role of interviewer discussed | Ethical approval obtained for study | Grounded theory used. Analysis and interviewing con-current. Full details given | None given |
| [Wisten and Zingmark (2007](#_ENREF_52)) | To elucidate perceived support and understand parents needs after sudden cardiac death | In –depth interviews with bereaved parents | Purposive sample of parents from study on sudden cardiac death | Not stated how interview schedule developed. Interview in parents’ home Interviews recorded and transcribed. | Not stated | Ethical approval obtained for study, ethical issues discussed. | Content analysis method used, 2 authors analysed data. | None given |
